# Knowledge and Attitudes of Teachers and School Professionals Toward Epilepsy: Results of an Educational Program in Greece

**DOI:** 10.7759/cureus.104703

**Published:** 2026-03-05

**Authors:** Stergiani Pitta, Angelos N Papadopoulos, Eirini Tsiamaki, Angeliki Tsapanou, Nikolaos Trimmis, Emilia Michou, Eleni Jelastopulu, Panagiotis Plotas

**Affiliations:** 1 Department of Speech and Language Therapy, School of Health Rehabilitation Sciences, University of Patras, Patras, GRC; 2 Department of Speech and Language Therapy, School of Health Sciences, University of Ioannina, Ioannina, GRC; 3 Department of Neurology, University General Hospital of Patras, Patras, Patras, GRC; 4 Department of Public Health, School of Medicine, University of Patras, Patras, GRC; 5 Laboratory of Primary Health Care, School of Health Rehabilitation Sciences, University of Patras, Patras, GRC

**Keywords:** education, educational program, education campaign, epilepsy, inclusive education, knowledge and attitudes, school teachers, seizure management, social stigma, training program

## Abstract

Background: The prejudices that accompany epilepsy in the school environment have negative consequences in the lives of children with epilepsy, such as stigmatization and marginalization. Many studies worldwide highlight that a significant percentage of teachers do not have the necessary knowledge about epilepsy and do not know what to do during an epileptic seizure. For this reason, the existence of educational programs for epilepsy among schoolteachers is suggested by these studies as a way forward. This urgent need to educate teachers about epilepsy has led several studies to focus on the planning and implementation of such health education programs.

Objectives: This study examined the impact of a specific educational program on epilepsy, conducted in Western Greece and aimed at teachers and school professionals in primary education. Five hundred and forty-five teachers and school professionals in primary education in Western Greece were administered to assess their knowledge and attitudes towards epilepsy. The results showed gaps in knowledge and negative attitudes. This led to the creation of a targeted educational program to examine its impact on changing teachers' knowledge and attitudes towards epilepsy.

Materials and methods: The study follows a quasi-experimental pre-post intervention design without a control group. The present study involved 471 primary school teachers, out of the 545 initially approached, who attended a targeted educational and interactive program on seizure education and management of epileptic seizures. Proposed up-to-date guidelines for epilepsy by the World Health Organization's action plan were considered. The material used for the presentation was evaluated by a Greek neurologist specializing in a university hospital and a general practitioner to present good first-aid practices for managing an epileptic seizure. The final training program was structured into two three-hour sessions, held over two consecutive days. After the training, the teachers were asked to complete the same questionnaire they had completed in the previous study. The data were analyzed with IBM SPSS Statistics software, version 28.0 (IBM Corp., Armonk, NY, USA). Limitations included potential selection bias and the absence of long-term follow-up.

Results: The findings indicate significant differences in respondents' answers before and after the intervention. More specifically, regarding knowledge about epilepsy, it seems that many participants after the intervention answered correctly questions related to the causes of epilepsy, the age at which it occurs, and the number of people it affects, as well as the existence of neurosurgery as a treatment option. Meanwhile, "I don't know" responses decreased significantly, shifting mainly towards the correct answers. In addition, knowledge about seizure management improved, as more participants answered correctly, incorrect responses decreased, and “I don’t know” responses in this area were almost eliminated. Furthermore, after the intervention, it seems that participants feel more confident in managing an epileptic seizure in the school environment.

Conclusions: The interactive training program had a positive impact on overall knowledge and attitudes; however, coordinated efforts by the entire educational community and health organizations are recommended to implement specialized training and guidance programs for teachers.

## Introduction

Epilepsy is a disease that is accompanied by many different misconceptions and prejudices worldwide [1-4]. Despite the clarification of the causes of this disease, some people still mistakenly believe that it is a psychiatric illness [4-7]. This has negative consequences in the lives of people with epilepsy, as they are often excluded from activities, stigmatized, and ultimately marginalized [2,5,8,9]. These negative consequences, however, often have different impacts depending on the age of the sufferers [10,11]. For example, an adult, who has different psychological defense mechanisms, may face it differently than a child, who has more difficulty managing his emotions. After all, childhood itself is a particularly sensitive period for a person's life [12], let alone for a child who has to manage a chronic condition such as epilepsy at the same time [13]. Therefore, the proper management of this situation from both the family and the school side is necessary and essential [14].

More specifically, as far as the school is concerned, proper information about the disease, understanding its causes and consequences in the lives of children who are ill, acceptance, and proper management of epileptic seizures [15] are just some of the necessary characteristics that teachers and school professionals should have so that they treat children who are ill equally and contribute in the best possible way to the management of the disease during school.

However, from theory to practice, there seems to be a big difference, since several studies [16-31], which have been conducted in recent years both at the European and global levels, show that teachers and school professionals have insufficient knowledge about epilepsy. At the same time, often their attitudes and behaviors can even be negative.

More specifically, approximately 20 years ago, a study by Bishop et al. (2006) in the USA revealed that many teachers had incomplete or incorrect knowledge about the causes of epilepsy, its symptoms, and the proper procedures for managing an epileptic seizure, despite holding positive attitudes [31].

In Africa, research by Berhe et al. in 2017 in Ethiopia showed that teachers had moderate knowledge and negative attitudes towards epileptic students, since prejudices influenced them [21]. The research by Toudou-Daouda & Ibrahim-Mamadouin (2020) in Nigeria showed that the most significant percentage had good or moderate knowledge; however, they lacked appropriate and correct techniques for dealing with epileptic seizures [22].

In Asia, similar results regarding teachers' knowledge and attitudes about epilepsy were shown by the research of Alkhamra et al. in 2012 in Jordan and Alqahtani in 2015 in Saudi Arabia [23,27]. In these studies, teachers' knowledge was characterized as moderate at both the theoretical and practical levels, while their attitudes were positive towards students who suffered [23,27].

In Europe, studies conducted in 2011 and 2014 by Mecarelli et al. in Italy demonstrated that teachers had insufficient knowledge about epilepsy and were cautious in providing first aid [24,28]. Furthermore, in the Czech Republic, the study by Brabcova et al. in 2012 showed that teachers had insufficient knowledge about seizure management, while their attitudes towards epileptic students were neutral or negative [26]. Similarly, in Germany, in 2025, the study by Kulawiak et al. showed that most teachers did not know how to manage an epileptic seizure [17]. In Spain, similar results for teachers' knowledge and attitudes towards epilepsy were shown by the study by Sansa et al. in 2021 [19]. Finally, in Greece, Toli et al., in 2013, showed with their research that many teachers could not recognize the manifestations of an epileptic seizure or an injury caused by it, while at the same time they used incorrect or even dangerous practices when providing first aid in an epileptic seizure [25]. Furthermore, the results of the research by Kampra et al. in 2016 showed that 81% (n=358) of teachers knew what epilepsy is and 89.9% (n=393) had a correct perception of the nature of the disease. However, 92.8% (n=413) did not have adequate training in the management of an epileptic seizure [29]. The recent study by Pitta et al. (2025) found that while most teachers were aware of epilepsy, they had gaps in their knowledge, as they did not know if it was a psychiatric disorder (33.5%, n = 19), and a significant percentage (55.4%, n = 302) reported an inability to manage an epileptic seizure, as well as to properly integrate epileptic students into the school environment [16].

The totality of all these studies worldwide highlights that a significant percentage of teachers do not have the necessary knowledge about epilepsy and do not know what to do during an epileptic seizure. For this reason, the existence of educational programs for epilepsy among school teachers is suggested by these studies as a way forward [16-27].

This urgent need to educate teachers about epilepsy has led several studies to focus on the planning and implementation of such health education programs [24,32-41]. In Turkey, the study by Kartal et al. evaluated the effectiveness of an epilepsy education program for primary school teachers, and the results showed a significant improvement in participants’ knowledge after the training [32]. In India, a study by Murthy et al. investigated the impact of an epilepsy education program on teachers and students. The results showed a significant improvement in knowledge, attitudes, and practices regarding epilepsy [34]. In Saudi Arabia, Alkhotani et al. conducted a study to evaluate the effects of health education on teachers’ first aid management in the event of a seizure. The results showed that when providing first aid in an epileptic seizure, 45.9% (n=119) of teachers initially stated that they would ensure the safety of the individual and seek help in the event of a seizure. Following the intervention, this percentage improved significantly, reaching 84.2% (n=218), representing a 38.2% increase (p = 0.001) [35]. In 2017, a study by Dumeier et al. investigated the effectiveness of a seizure management training program in Germany, focusing on practices such as rectal and buccal rescue medication administration. Results showed an increase in teachers’ confidence in administering rescue medication, as well as a reduction in errors during the administration process [33]. In Japan, Ozama et al. conducted a study that included a training program comprising theoretical instruction and practical practice in administering buccal midazolam. The study showed significant improvements in participants’ knowledge and confidence in seizure management and buccal midazolam administration, with participants reporting increased readiness and ability to respond to seizures in the school setting after the intervention [37]. Similar results have been reported regarding health education programs for teachers in studies conducted in various regions of Italy [24,37,39]. More specifically, Mecarelli et al. (2014) carried out an intensive and targeted educational program aimed at improving teachers' knowledge and attitudes. The program included a presentation on clinical manifestations of epilepsy, the distribution of informational leaflets, and an educational kit on the disease and its management. The results showed that the number of "do not know" answers was significantly reduced for almost all questions; however, negative attitudes remained [24]. Furthermore, in 2020, Renzetti et al. conducted a study aimed at educating school staff on the proper management of seizures, increasing safety, promoting the administration of rescue medications, and reducing inappropriate calls to the emergency number. The results showed an increase in knowledge about epilepsy, better knowledge about the management of seizures in the school environment, a decrease in anxiety, and an increase in willingness to administer rescue medications [36]. Finally, the study by Bert et al. investigated the effect of educational programs on school staff's management of seizures when administering rescue medications, aiming to enhance their knowledge, confidence, and preparedness [38].

In Greece, although some studies have been carried out to outline the knowledge, attitudes, and beliefs of primary and secondary education teachers [16,25,29,42] of public and private schools, no health education program has been implemented that addresses teachers and school professionals. However, the results of all these studies reveal both knowledge gaps and incorrect management practices in the treatment of epileptic seizures, which is why they recommend implementing health education programs in schools nationwide [16,25,29].

The current study is connected with the study “Teachers’ and School Professionals’ Knowledge and Attitudes Towards Epilepsy in Greece: Misconceptions and Management Options for Affected Students-A Survey Study” published in 2025 [16]. In that study, our research team attempted to record the knowledge, attitudes, and beliefs of 546 teachers and school professionals regarding epilepsy, who participated in the study and work in primary schools in Western Greece [16]. The participants were invited to answer a questionnaire derived from the Italian study “Knowledge and Attitudes toward Epilepsy among Primary and Secondary Schoolteachers in Italy” [28], after the relevant permission for use had been obtained from its creators and translation and linguistic correction had been carried out [16]. The final questionnaire used as a tool for this research was in a structured format consisting of six demographic questions and 28 questions that explored three main themes: (A) general and specific knowledge about epilepsy, (B) attitudes towards the social and individual impacts of epilepsy, and (C) attitudes related to school life [16]. The findings of our study reveal substantial gaps in the knowledge of primary school teachers and school professionals about epilepsy, while at the same time, highlighting various perceptions and behaviors that may influence their educational approach and lead to stigmatization and limitation of the full participation of students with epilepsy in school life. To address these problems, our research proposed the implementation of comprehensive training and health education programs for teachers through educational seminars and workshops that would provide basic information about epilepsy, reflect modern guidelines for physical activity of epileptic students, and offer practical training in the management of epileptic seizures [16].

Despite increasing international attention to epilepsy awareness in schools, structured intervention studies evaluating short-term educational impact on teachers’ knowledge, attitudes, and seizure first-aid preparedness remain limited in Greece. The present study addresses this gap by evaluating a structured interactive training program delivered to primary school teachers in Western Greece following a previously conducted baseline survey.

Considering the data from our previous research, as well as the studies from the global literature reviewed above that advocate for the implementation of health education programs for epilepsy, this study was conducted among teachers in school units. More specifically, the present study aimed to evaluate the short-term impact of a structured educational intervention on primary school teachers’ and school professionals’ knowledge about epilepsy, seizure first-aid competence, and stigma-related attitudes in the school setting. The study compares responses collected before the intervention (Phase A) and one week after training completion (Phase B).

## Materials and methods

Study design

The present study is a quantitative quasi-experimental pre-post intervention study without a control group, conducted among primary school teachers and school professionals. This study was carried out from September 2023 to April 2025 in Western Greece. Primary school teachers (both general and special education) and school professionals who had participated in Phase A of the research by answering the questionnaire (pre-test) of our previous research [16] were approached again to attend a comprehensive training program on epilepsy in the school units where they served. The same questionnaire was administered one week after completion of the training (post-test) in order to assess short-term changes in participants’ knowledge, attitudes, and first-aid practices.

Due to the lack of a comparison group and randomization, the causal conclusions drawn regarding the effectiveness of this intervention cannot be definitively established.

To properly design and organize the training program in detail for primary education teachers on epilepsy, proposals for creating interventions, training, and educational programs within school units were considered, drawing on the aforementioned global literature. In addition, modern guidelines for epilepsy proposed by the World Health Organization's action plan [42,43] were considered. The material used for the presentation was evaluated by a Greek neurologist specializing in a university hospital and a general practitioner to present good first-aid practices for managing an epileptic seizure. In addition, emphasis was placed on recording the basic misconceptions, incorrect knowledge, and negative attitudes that the participants held in the first phase of our research. Finally, an effort was made to consider the basic elements of adult education. In this program, the personal needs, qualities, and peculiarities of the participating teachers were mainly obstacles, such as reduced available time for attendance, reduced concentration due to working hours, different ages, different levels of education, and lack of motivation, since there was no motivation to participate beyond the knowledge itself.

The final training program was structured into two three-hour sessions, held over two consecutive days. On the first day, a PowerPoint-based lecture (Microsoft Corp., Redmond, WA, USA) was held that included information on the history of epilepsy, general information on the disease, its causes, its manifestations, its symptoms, its treatment, photographs with images of partial and generalized epilepsy, information on the cognitive and behavioral status of children with epilepsy, and finally, information related to everyday life (such as sleep, exercise, and mental health) and their quality of life, emphasizing social stigma and existing prejudices.

On the second day of the program, a presentation was initially made using images to identify various forms of epileptic seizures. The correct steps for providing first aid during the management of an epileptic seizure were then demonstrated, incorporating images and techniques, while incorrect and potentially dangerous manipulations were highlighted to avoid. At the end of the same day, the participants were invited to conduct practical training in the first aid techniques they had been taught, using hypothetical epileptic seizure scenarios in their school unit. The training was conducted as a group session, and after its completion, the participants received a pocket guide booklet providing instructions for the emergency management of an epileptic seizure and an infographic created by the Hellenic National Association against Epilepsy and the Panhellenic Scientific Association against Epilepsy, with correct and incorrect handling when providing first aid for the management of an epileptic seizure.

All training sessions were conducted by the same researcher, who was a school nurse with knowledge of adult education. A standardized curriculum and identical presentation materials (through PowerPoint slides, clinical real-life examples, and first aid demonstration scenarios) were followed to ensure consistency across all participating schools. The sessions were conducted using the same teaching structure and content outline.

**Figure 1:**
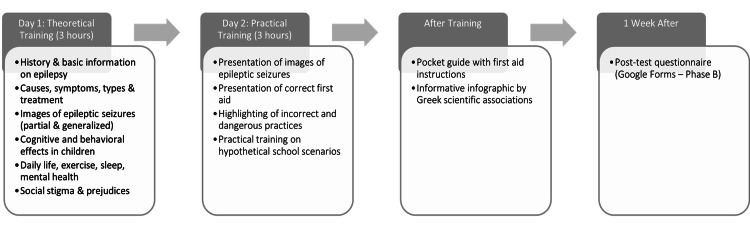
Flowchart of the Epilepsy Training Program

One week after the training activity, the same questionnaire that participants had completed in Phase A of the study was administered again to them in Phase B (post test) via Google Forms (Google Inc., Mountain View, CA, USA; Figure 1).

Study population

The research population consisted of all primary education teachers in general and special education who were serving in Western Greece during the conduct of this research.

Sample size

The 545 primary school teachers (general and special education) and school professionals who had participated in Phase A of the research by answering the questionnaire (pre-test) of our previous research [16] were invited again to attend a comprehensive epilepsy training program in the schools where they served (Phase B). Of these, 471 ultimately accepted to participate in Phase B of the research study and constitute its sample.

Study measures

The inclusion criteria were the same as those in our previous study [16], namely, participants had to be currently working as teachers or school professionals in primary education in Western Greece and have at least one year of teaching experience. The exclusion criteria included substitute teachers, part-time teachers, and individuals with a personal or family history of epilepsy, to minimize potential bias. Only fully completed questionnaires from both study phases were included in the final analysis. Incomplete responses were excluded from statistical testing.

Ethics statement

In order to comply with the basic rules governing a research study, permission was initially requested from the institutional body where the doctoral thesis is being carried out and from the supervising professor to conduct it. The study received ethical approval from the Bioethics Committee of the University of Patras, Patras, Greece (approval number 14787/24-11-2022). Furthermore, to protect and ensure the mental health of the participants in the study, all of them were informed about the research purpose of this study, that their participation is voluntary and anonymous, and that their answers will be used only for the benefit of the scientific and medical community. It was also clarified that participants can withdraw from the study during Phase A or B, or cancel their participation after completing the data. Furthermore, participation in the research was done with the consent of the subjects, who also provided written consent for their participation. Finally, the participating teachers were informed that the researcher's personal data would be disclosed to them in case they wished to be informed about the results of the study in which they participated.

Data collection instrument/questionnaire

The questionnaire used as a tool for this research to complete the second phase is the same as that used in our previous study and is a validated Greek-language version [16]. It consists of six demographic questions and 28 questions that investigate three main issues after the training: (A) general and specific knowledge about epilepsy, (B) attitudes towards the social and individual impacts of epilepsy, and (C) attitudes related to school life.

Statistical analysis

Results are presented with emphasis on clinically and educationally relevant outcomes, particularly seizure first-aid competence, confidence in management, and reduction of uncertainty responses. Descriptive statistics were performed for both quantitative and qualitative data. For the calculation of quantitative data, the mean and standard deviation were used, while for qualitative data, absolute and percentage frequencies were determined. Furthermore, an estimate of the frequencies was performed for the responses to each item of the questionnaire. Descriptive statistics are reported as numbers and percentages.

To examine the associations between categorical demographic variables (such as the gender of the participants, for example), the chi-square (χ²) test of independence was used. This test examined whether the distributions of the categorical variables differed significantly from one another. Differences between the groups that were re-examined were assessed using the χ² test. The change in the distribution of responses to each question between Phase A (i.e., the first stage of the survey) and Phase B (i.e., the second stage of the survey) was also assessed using the χ² test. Questions that elicited a correct answer were categorized as "Correct" versus "Incorrect" and were marked next to them with a symbol (✔) for correct. The significance level was set at α = 0.05 for all analyses. P-values less than 0.05 were considered statistically significant, while values equal to or greater than 0.05 were considered non-significant.

In addition to statistical significance testing, effect sizes were calculated for key pre-post comparisons using Cramér’s V in order to quantify the magnitude of observed differences. Effect sizes were interpreted according to conventional thresholds (small ≈0.10, medium ≈0.30, large ≥0.50).

The analysis of this research study aimed to examine the impact of an interactive educational program on primary school teachers and school professionals regarding epilepsy in terms of knowledge, attitudes, behaviors, and practices of dealing with an epileptic seizure during school (Phase B) in relation to previous research results obtained before the intervention (Phase A) in the same areas. IBM SPSS Statistics software for Windows 11, version 28.0 (IBM Corp., Armonk, NY, USA), was used for statistical analysis of the data.

Because of the large number of questionnaire items examined in this study, the possibility of inflated type I error due to multiple comparisons is important to consider when interpreting individual p-values. Therefore, results should be interpreted primarily in terms of overall patterns of change rather than through individual significant results.

## Results

Presented in detail below are the comparative results regarding the knowledge, attitudes, and behaviors of teachers towards epilepsy between Phase A (before the educational intervention) and Phase B (after the educational intervention). Statistical processing of the results was performed to facilitate descriptive comparisons and to assess the significance of all changes observed between the two phases. In some questions that were intended to evaluate the knowledge about epilepsy, the correct answers were specific and are indicated in the questionnaires with a check mark (✔). The statistical significance of all reported p-values is set at p < 0.05.

Demographic characteristics of the participants

Table 1 presents the basic demographic characteristics of the study participants. A total of 545 primary school teachers participated in Phase A of the study, while in Phase B, conducted after the health education intervention, 471 teachers completed the questionnaire. In Phase A, the average age of the participants was 40.61 years (SD = 10.32), and in Phase B, 41.52 years (SD = 10.90). The average years of teaching experience were 13.95 years (SD = 10.83) in Phase A and 14.29 years (SD = 10.81) in Phase B. Regarding gender distribution, 22.8% (124) of the participants in Phase A were male, while 77.2% (421) were female. In Phase B, the percentage of male participants decreased to 17% (80), while female participation increased to 83% (391).

**Table 1:** Baseline characteristics of the study’s participants ^1^Psychologist, Nursery, Speech therapist, Occupational therapist, Physiotherapist, Social worker, Special support staff; ^2^Philologist, Theologist, Art/Music teacher, Gym teacher, Informatics teacher, etc.

Variable	Phase A		Phase B	
	Mean	Standard Deviation	Mean	Standard Deviation
Age	40.61	10.319	41.52	10.897
Years of teaching	13.95	10.833	14.29	10.811
	n	%	N	%
Sex				
Male	124	22.8	80	17.0
Female	421	77.2	391	83.0
Experience in special education				
Yes	163	29.9	164	34.8
No	382	70.1	307	65.2
Attended courses on disabilities				
Yes	202	37.1	241	51.2
No	343	62.9	230	48.8
Specialty				
Primary school teacher	367	67.3	305	64.8
Special education teacher	28	5.1	41	8.7
Special education staff 1	97	17.8	92	19.5
Other 2	53	9.7	33	7.0

Personal knowledge of epilepsy before and after the intervention

Table 2 presents the personal knowledge of epilepsy before and after the intervention. Participants in both phases appeared to be very familiar with the term "epilepsy". Specifically, in Phase A, 541 of the 545 respondents (99.3%) reported knowing about epilepsy, compared to 470 of the 471 (99.8%) in Phase B. This increase was not statistically significant (χ² = 1.404, p = 0.236). This finding suggests that awareness of epilepsy was already high among participants prior to the intervention.

**Table 2:** Participants' knowledge of epilepsy χ²: chi-square

Questions	Phase A		Phase B		Comparison		
	n	%	N	%	x^2^		p
Do you know of a disease named epilepsy?					1.404		0.236
Yes	541	99.3	470	99.8			
No	4	0.7	1	0.2			
If yes, how?							
Have heard of it	300	55.0	247	52.4			
Personal/family experience	46	8.4	57	12.1			
From friends/acquaintances	81	14.9	92	19.5			
Discussion with doctors	58	10.6	70	14.9			
Scientific pamphlets	80	14.7	113	24.0			
Other	9	1.7	42	8.9			
Have you ever witnessed a seizure?					1.939		0.164
Yes	255	46.8	241	51.2			
No	290	53.2	230	48.8			
If yes, where?							
Classroom	79	14.5	73	15.5			
Public areas	104	19.1	132	28.0			
Home	14	2.6	20	4.2			
TV/Cinema	83	15.2	90	19.1			

Regarding the way in which they learned about epilepsy, the majority of participants in both phases stated that they had simply heard about the disease (Phase A: 55.0%, n=300; Phase B: 52.4%, n=247). However, the percentage of participants who reported personal or family experience with epilepsy increased from 8.4% (n=46) in Phase A to 12.1% (n=57) in Phase B.

Changes in attitudes toward epilepsy

Table 3 presents the changes in attitudes toward epilepsy. Teachers' knowledge of epilepsy showed a very significant improvement after the educational intervention. When participants were asked about their estimate of the incidence of epilepsy in Greece, those who gave the correct answer of 1 in 100 amounted to 10.1% (n=55) in Phase A and increased to 87.7% (n=413) in Phase B. The answer "I do not know" showed a large decrease from 74.7% (n=407) to 7.9% (n=37). The aforementioned differences were considered statistically significant (χ² = 590.988, p < 0.001).

**Table 3:** Participants' special knowledge of epilepsy ^1^ (χ^2^=621.640, p<0.001); ^2^ Right answers: A Phase: 2, B Phase: 193; χ²: chi-square

Questions	Phase A		Phase B		Comparison		Comparison (right answers)	
	n	%	n	%	x^2^	p	x^2^	p
How many people are affected by epilepsy in Greece?^1^					621.640	0.000	590.988	0.000
1/10	7	1.3	6	1.3				
✓ 1/100	55	10.1	413	87.7				
1/1000	63	11.6	14	3.0				
1/10000	13	2.4	1	0.2				
Don’t know	407	74.7	37	7.9				
What are the causes of epilepsy?^2^							268.653	0.000
✓ Genetics	172	31.6	434	92.1				
✓ Obstructed labor / Dystocia	55	10.1	401	85.1				
✓ Traumatic brain injury	79	14.5	445	94.5				
✓ Infections	55	10.1	259	55.0				
✓ Brain tumor	56	10.6	385	81.7				
Psychiatric disease	10	1.8	25	5.3				
All the above	21	3.9	0	0.0				
Don’t know	24	4.4	9	1.9				
What is the age of onset of epilepsy?					141.911	0.000	136.290	0.000
Childhood	52	9.5	10	2.1				
Adulthood	0	0.0	1	0.2				
✓ All ages	368	67.5	454	96.4				
Don’t know	125	22.9	6	1.3				
Do you think epilepsy is a form of psychiatric disease?					163.440	0.000	156.790	0.000
Yes	19	3.5	9	1.9				
✓ No	343	62.9	450	95.5				
Don’t know	183	33.6	12	2.5				
Do you think epilepsy is treatable with:							698.752	0.000
✓ Drugs	327	60.0	453	96.2				
✓ Neurosurgery	29	5.3	405	86.0				
Other	5	0.9	87	18.5				
Don’t know	201	36.9	10	2.1				
Do you consider epilepsy to be a curable disease?					370.259	0.000	352.805	0.000
Yes	131	24.0	39	8.3				
✓ No	178	32.7	427	90.7				
Don’t know	236	43.3	5	1.1				

In the questions concerning the causes of epilepsy, a significant increase was observed in the number of correct choices in Phase B compared to Phase A. For example, the correct identification of genetic factors increased from 31.6% (n=172) to 92.1% (n=434), obstructed labor from 10.1% (n=55) to 85.1% (n=401), traumatic brain injury from 14.5% (n=79) to 94.5% (n=445), infections from 10.1% (n=55) to 55.0% (n=259), and brain tumors from 10.6% (n=56) to 81.7% (n=385). Incorrect answers, such as “psychiatric illness” or “all of the above,” remained minimal and did not show a significant increase. The overall difference was also statistically significant (χ² = 268.653, p < 0.001).

In a question regarding the age of onset of epilepsy, participants who recognized that the condition could affect all ages constituted 96.4% (n=454) of participants in Phase B, compared to 67.5% (n=368) in Phase A. The percentage of respondents with doubts (“I don’t know”) decreased significantly from 22.9% (n=125) to 1.3% (n=6) (χ² = 136.290, p < 0.001).

A further improvement was observed in the percentage of respondents who recognized the lack of association between epilepsy and psychiatric illness, which rose from 62.9% (n=343) to 95.5% (n=450) in Phase B, while the “I don’t know” responses decreased from 33.6% (n=183) to 2.5% (n=12) (χ² = 156.790, p < 0.001).

Knowledge about treatment options also showed an impressive improvement. Awareness of drug therapy as a treatment method increased to 96.2% (n=453), and understanding of neurosurgery as a valid option increased from 5.3% (n=29) to 86.0% (n=405). These responses showed statistical significance, as did the reduction in individuals who did not know the answer (χ² = 698.752, p < 0.001).

Finally, the percentage of individuals who knew in Phase B that epilepsy is not curable was also significant, as was the reduction in the percentage of individuals who did not know this information (χ² = 352.805, p < 0.001).

Attitudes towards the social and individual burden of epilepsy

Table 4 presents the attitudes towards the social and individual burden of epilepsy. Teachers’ attitudes and perceptions regarding the limitations imposed by epilepsy and participation in physical activities also showed notable changes after the educational intervention. Their beliefs about the restrictions epilepsy may place on children’s physical activities at school were also affected. For example, a significantly higher percentage recognized driving as an activity limited to people with epilepsy, with rates increasing from 30.5% (n=166) in Phase A to 75.6% (n=356) in Phase B. The perception that epilepsy acts as a barrier to professional employment decreased significantly from 14.5% (n=79) to 3.2% (n=15), indicating a shift toward a less stigmatizing view. Other limitations, such as participation in sports activities, also showed a decrease, from 31.0% (n=169) to 16.6% (n=78) in Phase B. Moreover, the percentage of participants who believed that epilepsy does not entail any limitations fell from 23.1% (n=126) to 3.8% (n=18), suggesting a decline in generalized misconceptions.

**Table 4:** Attitudes towards the social and individual burden of epilepsy ^1^Right answers: A phase: 0, B phase: 202; χ²: chi-square

Questions	Phase A		Phase B		Comparison		Comparison	
	n	%	n	%	x^2^	p	x^2^	p
Do you think epilepsy limits:							-	-
Getting married	0	0.0	1	0.21				
Having children	5	0.9	3	0.64				
✓ Employment	79	14.5	15	3.18				
✓ Driving	166	30.5	356	75.58				
Sports activities	169	31.0	78	16.56				
None	126	23.1	18	3.82				
In your experience, recreational and sports activities of a child with epilepsy must be:					262.593	0.000	199.156	0.000
Normal	197	36.1	123	26.11				
✓ Limited	155	28.4	343	72.82				
Don’t know	193	35.4	5	1.06				
Which of the following sports do you think should not be recommended for a child with epilepsy?^ 1^							291.740	0.000
✓ Soccer	38	7.0	372	79.0				
Tennis	3	0.6	54	11.5				
✓ Swimming	104	19.1	393	83.4				
✓ Skiing	31	5.7	343	72.8				
Track and field events	63	11.6	70	14.9				
✓ Boxing	129	23.7	416	88.3				
Cycling	20	3.7	36	7.6				
All	36	6.6	11	2.3				
Don’t know	224	41.1	19	4.0				

At the same time, the percentage of participants who believed that recreational and sports activities for children with epilepsy should be limited increased from 28.4% (n=155) in Phase A to 72.8% (n=343) in Phase B. In this case as well, the response “I don’t know” showed a significant decrease, falling to just 1.1% (n=5), a statistically significant change (χ² = 199.156, p < 0.001).

Regarding sports considered unsuitable for children with epilepsy, participants increasingly identified activities that pose higher seizure-related risks. For example, soccer was considered inappropriate by 79.0% (n=372) of participants in Phase B, compared to only 7.0% (n=38) in Phase A. Similarly, skiing (from 5.7%, n=31 to 72.8%, n=343) and boxing (from 23.7%, n=129 to 88.3%, n=416) were also more frequently selected as unsafe after the intervention. Meanwhile, uncertainty was markedly reduced, with “I don’t know” responses decreasing from 41.1% (n=224) to 4.0% (n=19) (χ² = 291.740, p < 0.001).

Attitudes and practices related to epilepsy in the school environment

Table 5 presents the attitudes and practices related to epilepsy in the school environment. Participants' experiences and attitudes regarding epilepsy in the school setting showed significant improvement after the educational intervention. The percentage of teachers who had ever taught a student with epilepsy increased slightly from 29.0% (n=158) in Phase A to 38.0% (n=179) in Phase B. Among those with such experience, most had taught one or two students. Despite this experience, only 78.1% (n=368) of teachers in Phase B reported being informed by parents about a student’s epilepsy, a small decrease from 82.8% (n=451) in Phase A. However, there was a notable decrease in the percentage of teachers who did not know whether they had ever taught a student with epilepsy (from 7.5%, n=41, to 1.1%, n=5). The difference was statistically significant (χ² = 44.844, p < 0.001), indicating an improvement in communication practices between teachers, parents, and children, possibly aided by the intervention. A particularly significant effect was found in self-reported confidence in managing an epileptic seizure. While only 44.6% (n=243) of teachers in Phase A said they knew how to manage such a situation, this figure jumped to 92.1% (n=434) in Phase B (χ² = 257.950, p < 0.001), suggesting that the training dramatically improved perceived preparedness.

**Table 5:** School-life-related attitudes ^1^Right answers: A Phase: 0, B Phase: 287; χ²: chi-square

Questions	Phase A		Phase B		Comparison		Comparison	
	n	%	n	%	x^2^	p	x^2^	p
Have you ever had children with epilepsy in your classroom?					9.264	0.01		
Yes	158	29	179	38.0				
No	356	65.3	269	57.1				
Don’t know	31	5.7	23	48.9				
If yes. how many?								
One (1)	108	19.8	38	8.1				
Two (2)	23	4.2	6	1.3				
Three (3)	16	2.9	13	2.8				
More than 3	1	0.2	3	0.6				
Are you usually informed by the parents of a child’s epilepsy?					44.844	0		
Yes	451	82.8	368	78.1				
No	53	9.7	98	20.8				
Don’t know	41	7.5	5	1.1				
Do you know how to manage a child experiencing an epileptic attack?					257.95	0		
Yes	243	44.6	434	92.1				
No	302	55.4	37	7.9				
In the case of a seizure in class, what do you do?^1^							462.832	0
Call an ambulance	219	40.2	118	25.1				
✓ Have the child lie down and wait until the attack ends	174	31.9	456	96.8				
Place something in the child’s mouth	43	7.9	4	0.8				
Block the spasms of the limbs	58	10.6	7	1.5				
✓ Administer medications endorectally	51	9.4	355	75.4				
Don’t know	88	16.1	1	0.2				
In your school, are there difficulties in administering antiepileptic drugs during school hours?					229.584	0		
Yes	236	43.3	406	86.2				
No	46	8.4	36	7.6				
Don’t know	263	48.3	29	6.2				
In your opinion, does epilepsy impair children’s learning?					245.59	0	208.35	0
Always	18	3.3	7	1.5				
✓ Sometimes	216	39.6	396	84.1				
Never	82	15	52	11				
Don’t know	229	42.1	16	3.4				
In your opinion, do children with epilepsy require support in school?					148.678	0	132.669	0
Always	89	16.3	34	7.2				
✓ Sometimes	313	57.5	423	89.8				
Never	22	4	9	1.9				
Don’t know	121	22.2	5	1.1				
In your opinion, do children with epilepsy have mental disorders?					442.16	0	418.58	0
Always	3	0.6	2	0.4				
✓ Sometimes	149	27.2	429	91.1				
Never	136	25	40	8.5				
Don’t know	257	47.2	0	0				
In your opinion, do children with epilepsy have behavioral disorders?					294.346	0	284.077	0
Always	15	2.8	5	1.1				
✓ Sometimes	217	39.8	428	90.8				
Never	87	16	25	5.3				
Don’t know	226	41.4	13	2.8				
In your opinion, to what extent do antiepileptic drugs affect learning?					226.592	0	171.226	0
Always	18	3.3	9	1.9				
✓ Sometimes	219	40.2	380	80.7				
Never	65	11.9	60	12.7				
Don’t know	243	44.6	22	4.7				
In your opinion, to what extent do antiepileptic drugs affect behavior?					273.527	0	94.348	0
Always	13	2.4	5	1.1				
Sometimes	198	36.3	260	55.2				
✓ Never	73	13.4	189	40.1				
Don’t know	261	47.9	17	3.6				
In your opinion, do children with epilepsy have relationship problems with their peers?					228.398	0	125.528	0
Always	11	2	5	1.1				
✓ Sometimes	244	44.8	373	79.1				
Never	55	10.1	80	17				
Don’t know	235	43.1	13	2.8				
Compared with their classmates, how should children with epilepsy be treated?					71.764	0		
Same	400	73.4	438	93				
Differentiated	103	18.9	31	6.6				
Don’t know	42	7.7	2	0.4				
How should the demands on children with special needs be compared to those on their classmates?					129.383	0		
Same	263	48.3	385	81.7				
Differentiated	236	43.3	83	17.6				
Don’t know	46	8.4	3	0.6				
According to your experience, how do classmates behave toward a child with epilepsy?					20.18	0		
Normally	243	44.6	236	50.1				
Try to help	161	29.5	165	35				
Tend to marginalize	44	8.1	16	3.4				
Don’t know	97	17.8	54	11.5				

Another positive finding was that responses to questions regarding teachers’ management strategies during a seizure shifted in more positive directions. Only 31.9% (n=174) of teachers in Phase A said they would allow the child to lie down and wait for the seizure to pass, compared to an impressive 96.8% (n=456) in Phase B. Similarly, the percentage of teachers who would choose to administer medication rectally increased from 9.4% (n=51) to 75.4% (n=355). Harmful misconceptions, such as placing an object in the child’s mouth or restricting limb movement, decreased significantly (from 7.9%, n=43, to 0.8%, n=4, and from 10.6%, n=58, to 1.5%, n=7, respectively). These findings are representative of both theoretical and practical improvements in the management of emergencies in the school environment. Regarding the administration of antiepileptic drugs in school, teachers in Phase B were much more aware of the existence of difficulties, with affirmative responses increasing from 43.3% (n=236) to 86.2% (n=406) (χ² = 229.584, p < 0.001) and “don’t know” responses decreasing significantly, suggesting that teachers gained increased awareness of institutional constraints (Table 6).

**Table 6:** Effect size analysis of key knowledge outcomes χ²: chi-square

Variable	Phase A (%)	Phase B (%)	Risk Difference (B–A)	χ²	Cramer’s V
Correct estimation of epilepsy prevalence (1/100)	10.1	87.7	77.60%	590.988	0.763
Correct recognition that epilepsy is not a psychiatric disorder	62.9	95.5	32.60%	156.79	0.393
Awareness of neurosurgery as a treatment option	5.3	86	80.70%	698.752	0.829
Correct recognition that epilepsy is not curable	32.7	90.7	58.00%	352.805	0.589
Recognition that epilepsy may sometimes affect learning	39.6	84.1	44.50%	208.35	0.452

A similar trend was observed in changing beliefs about the impact of epilepsy and its treatment. A greater proportion of teachers were aware that epilepsy can sometimes affect learning (up from 39.6%, n=216, to 84.1%, n=396) or behavior (39.8%, n=217, to 90.8%, n=428), and that antiepileptic drugs can occasionally have consequences in both areas (e.g., 40.2%, n=219, to 80.7%, n=380 for learning). The reduction in “don’t know” responses across all items was striking and consistent. Finally, attitudes towards school inclusion improved, with more teachers agreeing that students with epilepsy should be treated like all students (up from 73.4%, n=400 to 93.0%, n=438) and that educational requirements should not be differentiated (48.3%, n=263 to 81.7%, n=385). Even peer attitudes towards people with epilepsy improved greatly as the number of teachers who stated that students with epilepsy are marginalized by their classmates decreased (Table 7).

**Table 7:** Effect size analysis of seizure management and school practice outcomes χ²: chi-square

Variable	Phase A (%)	Phase B (%)	Risk Difference (B–A)	χ²	Cramer’s V
Self-reported confidence in seizure management	44.6	92.1	47.50%	257.95	0.504
Correct positioning (allowing the child to lie down)	31.9	96.8	64.90%	462.832	0.675
Willingness to administer rectal medication	9.4	75.4	66.00%	462.832	0.675
Agreement that children with epilepsy should be treated equally	73.4	93	19.60%	71.764	0.266

## Discussion

This study aimed to examine the impact of a specific educational program on epilepsy conducted in Western Greece, which is addressed to teachers and school professionals in primary education. The observed improvements should be interpreted as educationally meaningful short-term changes rather than definitive causal intervention effects. As shown in the statistical data, there are significant differences between the respondents' answers before and after the intervention. More specifically, regarding knowledge about epilepsy, it seems that many participants after the intervention answered correctly questions related to the causes of epilepsy, the age at which it occurs, and the number of people it affects, as well as the existence of neurosurgery as a treatment option. Meanwhile, the "I don't know" responses decreased significantly, shifting mostly towards the correct answers. In addition, knowledge about seizure management improved, as more participants answered correctly, incorrect responses decreased, and “I don’t know” responses in this area were almost eliminated. Furthermore, after the intervention, it seems that participants feel more confident in managing an epileptic seizure in the school environment. Other international studies show similar results. More specifically, in the survey by Sulena et al., which investigated the effect of an educational intervention on teachers’ knowledge, attitudes, and practices regarding epilepsy in India, the results showed that after the intervention, there was a significant improvement in participants’ knowledge, attitudes, and practices regarding epilepsy, including recognizing the characteristics of seizures and providing proper first aid [18]. The same results were also shown after the intervention regarding the provision of first aid in an epileptic seizure in the study of Alkhotani et al. in Saudi Arabia [35]. Furthermore, the results of the interventions carried out on Italian teachers in the studies of Renzetti et al. and Mecarelli et al. showed, on the one hand, an increase in the readiness of teachers to manage epileptic seizures and administer rescue medications and, on the other hand, a reduction in the responses "I don't know," but a limited change in attitudes containing specific prejudices about the disease [24, 36]. Furthermore, in the study of Dumeier et al., it appears that after the intervention, the self-confidence of the participants also increased from 5 to 8 on a scale of 1 to 10 (p<0.001) [33]. The results of these surveys are fully aligned with the results of the present study.

It is obvious that the training program also had a positive effect on the axis of school life and performance of children with epilepsy, since the participants answered most of the questions correctly, while at the same time, the number of "I don't know" answers decreased. However, a big statistical difference seems to be presented by the questions regarding the sports allowed for children with epilepsy. Specifically, 343 participants after the intervention believe that children with epilepsy should have limited sports activity, compared to 155 who were before the training action. Furthermore, it seems that most teachers, after the intervention, are oriented towards the fact that children with epilepsy should refrain from activities with an increased risk of injury, such as football, swimming, skiing, and boxing. The increase in this percentage may be due to the better information of teachers about the disease of epilepsy, which results in more responsibilities. It is obvious that, although the training program that the teachers attended regarding epilepsy had as its ultimate goal the demystification of the disease and the better integration of the students in the school environment, the trainee teachers and school professionals were faced with the responsibility that the knowledge they received gave rise to, which possibly also affects their attitudes, especially on issues related to physical safety. Corresponding research that has been carried out in the same field has shown that, after training programs, teachers are often more cautious about allowing students with epilepsy to participate in high-risk sports, such as boxing and swimming, without the necessary supervision [18]. Of course, this fact should not concern us, since their reticence is no longer based on prejudices but on an enhanced fear that stems from the responsibility and legal consequences that may arise in the event of an epileptic episode or accident. In fact, in countries such as Greece, where the legislative framework is not entirely clear regarding the obligations and limits of responsibility of teachers in such critical health situations, teachers are often led to overprotective behaviors towards their students, limiting their possibilities of participating in certain actions. Therefore, in order to change this attitude, there should be certain amendments to the legislation in countries such as Greece, where there are gaps and ambiguities regarding the obligations and limits of involvement of teachers and school professionals in cases of health emergencies in school units. Furthermore, a national updated scientific guide with guidelines should be created, related to sports and activities that are allowed or avoided in certain chronic diseases, which should be distributed to all regular and special education schools to facilitate the resolution of such dilemmas and reduce prejudices regarding specific chronic diseases by teachers. Moreover, according to the guidelines of the International League Against Epilepsy (ILAE) and the World Health Organization, physical activity of epileptic students should be encouraged, since it contributes positively to both the psychosocial development of the child and their good physical health [43].

Another striking statistical difference worth discussing is that 86.2% (n=406) of respondents after the training believe that there are difficulties in administering antiepileptic drugs at school, compared to 43.3% (n=236) who believed this before the training. This percentage may have arisen due to the presentation of the legislative framework that currently exists in Greece and governs the school life of teachers during the first meeting of the training program held for teachers. More specifically, the legislation in force to date clarifies that "teachers are not required to have medical or pharmaceutical knowledge in order to administer medication, but they must provide basic first aid to students in need of assistance. Furthermore, in emergencies, they must call the emergency number and inform the parents of the affected students". The prefecture also states that "there is a need for cooperation between school units and medical services"; however, it does not provide any information on how this can be done, nor does it provide clear guidelines for direct intervention by teachers in critical health situations. Finally, the legislative framework in Greece does not clearly state the responsibilities of teachers in cases of epileptic seizures [44]. This legislative ambiguity creates a strong climate of insecurity among teachers and school professionals and often prevents them from providing additional assistance or taking the initiative in crisis situations. Even those who have received training in certain first aid techniques do not apply them for fear of the legal consequences that follow if something goes wrong. To overcome this responsibility-averse attitude of teachers, on the one hand, there should be a clear legal enshrining of the roles and limits of responsibility that each educational specialty has within the school unit in cases of emergency health issues. In addition, the existence of predefined protocols for dealing with health crises, with specific, simple, and clear instructions for each incident, would contribute even more to eliminating the fear of administering basic life-support medications, such as antiepileptic drugs. Finally, it would be useful to have organized regular training in legal issues and ethical dilemmas that govern school life, through nationally approved legal education programs, so that there are no wrong impressions and misinterpretations regarding the reading of the law.

Considering the above, it seems that the intervention was quite successful and constructive, since it managed to improve both the knowledge and attitudes of the participants about epilepsy and to significantly reduce the number of people who did not know enough about it and, by extension, contributed to the correct provision of first aid during an epileptic seizure. In other words, it seemed that this combination of different means and training methods worked particularly constructively in the sample of participating teachers. More specifically, the use of images contributed to the visualization of situations of epileptic seizure and epilepsy, and contributed to their better understanding. In addition, the practical training with the practice in realistic incidents increased the self-confidence and preparedness of the trainees while contributing to their emotional involvement, which, as it seemed, led to a change in attitudes. Finally, the discussion and resolution of personal questions contributed to the reduction of misunderstandings and the increase in the participants' knowledge. After all, according to Knowles' theory, adult education is best achieved through active learning [45]. According to Kolb's Experiential Learning Cycle (Experience, Reflect, Conceptualize, Apply), it appears that "doing" is superior to "listening" or "reading" and leads to better learning outcomes [46]. This fact should be taken seriously when designing and implementing education policies to create programs on an annual basis so that teachers are informed about their students' health issues, such as epilepsy, but also learn actively through their participation in possible realistic scenarios how to properly deal with health emergencies within the school unit.

Our research highlights that the implementation of comprehensive training programs for teachers on health issues such as epilepsy has a positive impact on improving their knowledge, attitudes, and behaviors towards children who are affected, which also contributes to better educational treatment and ultimately to a better quality of life. This fact reinforces the need to organize educational seminars and workshops for teachers, students, and parents, which provide modern guidelines on epilepsy and practical training in seizure management. Comparisons with other studies, both in Greece and internationally, confirm that although knowledge about epilepsy is incomplete and the problem is widespread, teacher training programs have all had positive results in both informing and reducing the stigmatization of children with epilepsy, which is why the elimination of the phenomenon requires coordinated action.

The Global Action Plan on Epilepsy and Other Neurological Disorders 2022-2031 (IGAP) proposed by the World Health Organization has exactly this goal, namely the strengthening of health services and the promotion of research and innovation on issues such as epilepsy, through collaboration between various bodies, the development of national strategies, the revision or development of appropriate legislation to protect the rights of people with epilepsy, and raising public awareness about neurological disorders [47]. Greece, as a WHO member state, is equally committed to implementing the IGAP project, which means that it should adapt its policies and legislative framework regarding epilepsy to promote a supportive and inclusive environment for people with epilepsy.

The development of technology is another encouraging factor that can contribute positively to the design and implementation of training programs on health issues for teachers. It is obvious that each person can learn in a different way and at a different time; therefore, better knowledge and change of attitudes can only be achieved through the individualization of needs. With the use of artificial intelligence, adaptive platforms of targeted learning could be created, which would have the ability, through quizzes to identify weak points in the knowledge and attitudes of teachers on health issues, such as epilepsy, and to adapt the training accordingly so as to serve the educational needs of each teacher in order to improve them or even eliminate the problem. Furthermore, artificial intelligence could contribute to the creation of interactive training with virtual epileptic students so that teachers are called upon through realistic simulation scenarios to recognize different types of epileptic seizures and are trained in real time in providing first aid without fear and anxiety.

Although the training seems quite successful, the present study has some strengths and limitations. Its main strength is the large number of participants and the representativeness of the sample, since in Phase B of the study, the number of participants was 471 teachers working during this school year, a number greater than the 382 participants that would have been necessary, with an acceptable margin of error of 5% and a confidence level of 95%. Although the large preponderance of female participants limits the generalizability of the findings, this result is in line with the data prevailing in Greek primary education, which is mainly female-dominated. However, the first important limitation is the large lack of male participants, which limits the generalizability of the findings to a wider educational population. A second limitation is the geographical area of Western Greece, which limits the application of the results to other regions of Greece. A third major limitation is the type of questions used in the questionnaire, which may allow for different interpretations by participants and could lead to inaccuracies in the responses. A fourth major limitation is that the participants who chose and are participating in phase B may be more aware of epilepsy and their students, and, as a result, were better in their responses compared to the average teacher. A fifth limitation is the place and time of the training program, which were not always the same. This could result in different performance and training opportunities, both on the part of the trainees and on the part of the trainer-researcher. More specifically, the size of the space, the level of external noise, the fatigue of the teachers (especially if the meeting was immediately after school hours), the mood of the researcher, the distance and accessibility of the school unit (remote areas were more difficult to access, which caused fatigue for the speaker), etc. An important limitation is the absence of a control group; therefore, causal inference regarding the effectiveness of the intervention cannot be definitively established. In addition, the pre-post design evaluated short-term changes only, and no long-term follow-up data were available to assess knowledge retention over time. Despite the relatively large sample, comparisons between subgroups were not possible due to small numbers in each category of teacher specialty. Finally, because multiple questionnaire items were tested statistically, the possibility of inflated type I error should be considered when interpreting individual significant findings.

## Conclusions

This study demonstrated substantial short-term improvements in teachers’ epilepsy knowledge, confidence, and self-reported seizure first-aid practices following a structured educational intervention. Future research should focus on different aspects than those that have already been analyzed from time to time, with the aim of expanding and deepening the understanding of epilepsy. First, more qualitative studies are needed, which explore in depth students' views and feelings about how they experience the situation of their school life in relation to the existence of their illness, that is, to analyze the areas in which they often face problems of inclusion or exclusion. Second, to carry out studies that simultaneously involve teachers, parents, and students, so that there is a holistic picture of epilepsy in schools. Third, to create and develop more balanced and reliable tools, such as questionnaires, to lead to more in-depth results and to the study of different factors that influence this specific issue. Fourth, to carry out comparative studies between different regions of Greece and other countries with different cultural beliefs and geographical distributions, which may affect the knowledge and perceptions of teachers. Fifth, comparative studies with different techniques for training teachers on health issues, to highlight the shortest and most efficient one, so that it can be widely used in such training cases. Finally, future research could deal with the creation and integration of more modern means of communication and information on epilepsy and other neurological diseases, such as the construction of an application capable of providing an interface between teachers and all agencies for collaboration and recording of health emergency cases that occur in school units in real time.

It is obvious that cooperation between countries, but also between institutions, is the only way to address entrenched attitudes and perceptions and to eliminate prejudices about epilepsy. Therefore, in a modern world with intense technological and scientific discoveries, it is impossible for states to lag behind developments by maintaining education systems with outdated knowledge and perceptions, which stigmatize and marginalize, as well as relying on legislation that does not defend and protect its citizens in matters of safety and health.
